# Post-weaning selenium and folate supplementation affects gene and protein expression and global DNA methylation in mice fed high-fat diets

**DOI:** 10.1186/1755-8794-6-7

**Published:** 2013-03-05

**Authors:** Emma N Bermingham, Shalome A Bassett, Wayne Young, Nicole C Roy, Warren C McNabb, Janine M Cooney, Di T Brewster, William A Laing, Matthew PG Barnett

**Affiliations:** 1 Food Nutrition & Health Team, Food & Bio-based Products Group, AgResearch Grasslands, Palmerston North 4442, New Zealand; 2 Riddet Institute, Massey University, Palmerston North 4442, New Zealand; 3 AgResearch Grasslands, Palmerston North, 4442 New Zealand; 4 Biological Chemistry & Bioactives, Food Innovation, Plant & Food Research Ruakura, Hamilton 3240, New Zealand; 5 Biological Chemistry & Bioactives, Food Innovation, Plant & Food Research Mt Albert, Auckland 1025, New Zealand

**Keywords:** Epigenetic, Microarray analysis, 2D-DIGE, Proteomics, Folate, Selenium, High fat

## Abstract

**Background:**

Consumption of high-fat diets has negative impacts on health and well-being, some of which may be epigenetically regulated. Selenium and folate are two compounds which influence epigenetic mechanisms. We investigated the hypothesis that post-weaning supplementation with adequate levels of selenium and folate in offspring of female mice fed a high-fat, low selenium and folate diet during gestation and lactation will lead to epigenetic changes of potential importance for long-term health.

**Methods:**

Female offspring of mothers fed the experimental diet were either maintained on this diet (HF-low-low), or weaned onto a high-fat diet with sufficient levels of selenium and folate (HF-low-suf), for 8 weeks. Gene and protein expression, DNA methylation, and histone modifications were measured in colon and liver of female offspring.

**Results:**

Adequate levels of selenium and folate post-weaning affected gene expression in colon and liver of offspring, including decreasing *Slc2a4* gene expression. Protein expression was only altered in the liver. There was no effect of adequate levels of selenium and folate on global histone modifications in the liver. Global liver DNA methylation was decreased in mice switched to adequate levels of selenium and folate, but there was no effect on methylation of specific CpG sites within the *Slc2a4* gene in liver.

**Conclusions:**

Post-weaning supplementation with adequate levels of selenium and folate in female offspring of mice fed high-fat diets inadequate in selenium and folate during gestation and lactation can alter global DNA methylation in liver. This may be one factor through which the negative effects of a poor diet during early life can be ameliorated. Further research is required to establish what role epigenetic changes play in mediating observed changes in gene and protein expression, and the relevance of these changes to health.

## Background

The epigenome comprises interconnected, interdependent heritable processes (including DNA methylation, histone modifications, and non-coding RNAs) that regulate gene expression in response to environmental influences. Epigenetic regulation may be as important as DNA transcription and translation for allowing plasticity of the phenotype in a fixed genotype [1]. There is mounting evidence that epigenetics has a major role in multiple physiological processes including development, imprinting, and regulation of gene transcription [2]. Activation or deactivation of genes in response to various deleterious environmental stimuli may be important in the development of common complex diseases [3,4]. Specific nutrients can modulate epigenetic events [5,6], indicating that diet may be a key environmental factor which can influence epigenetic patterns [7,8], with consequent effects on gene expression. It is therefore important to investigate the potential of food components to induce phenotypic changes via epigenetic modulation of gene expression [9].

A genetic predisposition to good health may be established at conception by the combination of genetic material from both parents [10]. However, the effects of environmental impacts throughout life, particularly during critical times such as gestation, can influence the expression of the genetic potential of the individual, resulting in altered phenotype [10]. Macronutrient-rich diets high in carbohydrate, protein and fat may have inadequate levels of micronutrients, especially if the food components are heavily processed [11]. Poor micronutrient intake, which is often associated with high consumption of such foods, has been linked to late onset diseases such as cancer [12]. Selenium is an essential micronutrient which is deficient in diets in several countries, including New Zealand [13], and this deficiency can result in symptoms associated with oxidative stress [14]. Folate, a water-soluble B vitamin with roles in DNA synthesis and repair, is also nutritionally inadequate in diets in many countries [15]. Both selenium and folate can influence gene expression, possibly via epigenetic mechanisms [16]. Folate is thought to affect epigenetic regulation via one-carbon metabolism, however the epigenetic regulation by selenium is less well understood [17]. Selenium is thought to modulate folate deficiency, suggesting that the interaction between folate and selenium is important [16].

Epigenetic activation and repression is important in normal cellular development and maintenance [18]. While particularly important during foetal development, these processes have also been implicated in postnatal development, affecting for example both structure and function of the mammalian gastrointestinal tract [19]. Histone modification and DNA methylation are major epigenetic mechanisms [20]. In the case of methylation, the general dogma suggests that the addition of methyl groups by DNA methyltransferase represses gene expression, while methyl removal allows activation [21]. Dysregulation of these processes may lead to negative impacts on health [22]. Histone modifications include both acetylation and deacetylation (amongst other modifications); DNA methylation and histone modifications can act together to provide stable and heritable gene silencing in eukaryotic genomes [23].

We hypothesised that post-weaning supplementation with adequate levels of selenium and folate after exposure (via maternal diet during gestation and lactation) to high fat and low selenium and folate will lead to epigenetic changes in the offspring which are of importance for long-term health, with a high-fat diet being a risk factor for obesity. Of interest are the long-term effects of intergenerational epigenetic regulation, which are more likely to be carried through the maternal line [24]. To test this hypothesis, female mice were fed a high-fat diet containing low levels of selenium and folate during gestation and lactation. At four weeks of age, female offspring were either weaned onto the same diet (HF-low-low), or onto a high-fat diet containing adequate selenium and folate (HF-low-suf). At 12 weeks of age, gene and protein expression profiles, and DNA methylation (global and gene-specific) were measured in colon and liver, and selenium content and histone H3 methylation and acetylation measured in the liver. These analyses showed changes in gene and protein expression in metabolic and oxidative pathways, and altered global DNA methylation, due to post-weaning supplementation with selenium and folate.

## Results

### Food intake and body weight

There was no effect of adequate levels of selenium and folate post-weaning on food intake (c. 2.7 g diet/day; P = 0.91) or bodyweight (c. 18.5 g; P = 0.66) of offspring.

### Liver selenium content

There was a small but significant effect of diet on liver selenium, with levels in mice in the HF-low-suf group being slightly higher than those in mice in the HF-low-low group (1.03 ± 0.12 *vs.* 0.85 ± 0.15 mg/kg, P < 0.05).

### Gene and protein expression profiles

Post-weaning supplementation with adequate levels of selenium and folate altered the expression (P < 0.01, |fold change| >1.5) of 23 genes in the colon (Additional file 1: Table S1). The majority of these genes were significantly over-represented in Gene Ontology (GO) biological processes related to cellular and DNA metabolic processes (Table 1). The biological process “Lipid homeostasis” was also significantly over-represented (P = 0.007) among the differentially expressed genes in the colon. The mRNA abundance of the majority of genes in the colon was decreased (Table 1).

**Table 1:** Significantly over-represented gene ontology biological processes associated with differentially expressed genes in the colon of mice supplemented with adequate levels of selenium and folate post-weaning (n = 6 per treatment)

**Term**	**GOid**	**Gene name**	**Systematic name**	**Description**	**Fold change**	**P value**
Organelle organization	GO:0006996	Top2a	NM_011623	Mus musculus topoisomerase (DNA) II alpha	−1.7	0.006
		Stx6	NM_021433	Mus musculus syntaxin 6	−1.6	0.003
		Col4a3bp	NM_023420	Mus musculus procollagen, type IV, alpha 3 (Goodpasture antigen) binding protein	−1.5	0.004
		Pom121	NM_148932	Mus musculus nuclear pore membrane protein 121	−1.7	0.004
		Shprh	AK037266	Mus musculus 16 days neonate thymus	−1.6	0.003
Lipid homeostasis	GO:0055088	Col4a3bp	NM_023420	Mus musculus procollagen, type IV, alpha 3 (Goodpasture antigen) binding protein	−1.5	0.004
		Plscr3	NM_023564	Mus musculus phospholipid scramblase 3	−1.6	0.007
Cellular component organization	GO:0016043	Nov	NM_010930	Mus musculus nephroblastoma overexpressed gene	−1.7	0.005
		Top2a	NM_011623	Mus musculus topoisomerase (DNA) II alpha	−1.7	0.006
		Stx6	NM_021433	Mus musculus syntaxin 6	−1.6	0.003
		Col4a3bp	NM_023420	Mus musculus procollagen, type IV, alpha 3 (Goodpasture antigen) binding protein	−1.5	0.004
		Pom121	NM_148932	Mus musculus nuclear pore membrane protein 121	−1.7	0.004
		Shprh	AK037266	Mus musculus 16 days neonate thymus	−1.6	0.003
Nuclear pore organization	GO:0006999	Pom121	NM_148932	Mus musculus nuclear pore membrane protein 121	−1.7	0.004
Cellular process	GO:0009987	Heph	NM_181273	Mus musculus hephaestin, transcript variant 2	−1.5	0.001
		Mef2a	NM_001033713	Mus musculus myocyte enhancer factor 2A	−1.6	0.001
		Nov	NM_010930	Mus musculus nephroblastoma overexpressed gene	−1.7	0.005
		Rora	AK087905	Mus musculus 2 days pregnant adult female ovary	−1.8	0.001
		Top2a	NM_011623	Mus musculus topoisomerase (DNA) II alpha	−1.7	0.006
		Mtmr1	NM_016985	Mus musculus myotubularin related protein 1	−1.6	0.008
		Stx6	NM_021433	Mus musculus syntaxin 6	−1.6	0.003
		Col4a3bp	NM_023420	Mus musculus procollagen, type IV, alpha 3 (Goodpasture antigen) binding protein	−1.5	0.004
		Pom121	NM_148932	Mus musculus nuclear pore membrane protein 121	−1.7	0.004
		Shprh	AK037266	Mus musculus 16 days neonate thymus	−1.6	0.003
		Podxl2	NM_176973	Mus musculus podocalyxin-like 2	−1.6	0.005
DNA packaging	GO:0006323	Top2a	NM_011623	Mus musculus topoisomerase (DNA) II alpha	−1.7	0.006
		Shprh	AK037266	Mus musculus 16 days neonate thymus	−1.6	0.003
DNA conformation change	GO:0071103	Top2a	NM_011623	Mus musculus topoisomerase (DNA) II alpha	−1.7	0.006
		Shprh	AK037266	Mus musculus 16 days neonate thymus	−1.6	0.003
DNA metabolic process	GO:0006259	Top2a	NM_011623	Mus musculus topoisomerase (DNA) II alpha	−1.7	0.006
		Shprh	AK037266	Mus musculus 16 days neonate thymus	−1.6	0.003

A positive fold change indicates that supplementation increased the expression of the gene.

In the liver, post-weaning supplementation with adequate levels of selenium and folate led to differential expression (P < 0.01, FC > 1.5) of 36 genes (Additional file 2: Table S2), within which the GO biological processes, “Cellular process”, “Multicellular organismal process”, “Developmental process”, and “Multicellular organismal development” were significantly over-represented (P < 0.05) (Table 2). Whilst the mRNA abundance of the majority of these genes, including *Slc2a4*, was decreased, the abundance of *Dmbx1*, *Errfi1*, *Plce1* and *Fpr-rs2* transcripts was increased in the liver (Table 2) in offspring supplemented with adequate levels of selenium and folate.

**Table 2:** Significantly over-represented gene ontology biological processes associated with differentially expressed genes in the liver of mice supplemented with adequate levels of selenium and folate post-weaning (n = 6 per treatment)

**Term**	**GOid**	**Genename**	**Systematicname**	**Description**	**Fold change**	**P value**
Multicellular organismal process	GO:0032501	Il2	NM_008366	Mus musculus interleukin 2	−1.50	0.010
		Isl1	NM_021459	Mus musculus ISL1 transcription factor, LIM/homeodomain	−1.59	0.007
		Mbp	NM_010777	Mus musculus myelin basic protein, transcript variant 7	−1.62	0.004
		Ift172	NM_026298	Mus musculus intraflagellar transport 172 homolog (Chlamydomonas)	−1.52	0.002
		Errfi1	NM_133753	Mus musculus ERBB receptor feedback inhibitor 1	1.73	0.000
		Dmbx1	NM_130865	Mus musculus diencephalon/mesencephalon homeobox 1, transcript variant 1	−1.50	0.010
		Mcoln3	AK033008	Mus musculus 12 days embryo male wolffian duct includes surrounding region cDNA	−1.68	0.008
		Ndrg4	NM_145602	Mus musculus N-myc downstream regulated gene 4	−1.55	0.009
		Frem2	NM_172862	Mus musculus Fras1 related extracellular matrix protein 2	−1.52	0.007
		Olfr1355	AF042360	Mus musculus clone OR27-3 putative olfactory receptor mRNA	−1.53	0.001
		Olfr357	NM_146623	Mus musculus olfactory receptor 357	−1.52	0.008
		Olfr646	NM_147056	Mus musculus olfactory receptor 646	−1.67	0.010
Developmental process	GO:0032502	Il2	NM_008366	Mus musculus interleukin 2	−1.50	0.010
		Isl1	NM_021459	Mus musculus ISL1 transcription factor, LIM/homeodomain	−1.59	0.007
		Mbp	NM_010777	Mus musculus myelin basic protein, transcript variant 7	−1.62	0.004
		Slc2a4	NM_009204	Mus musculus solute carrier family 2 (facilitated glucose transporter), member 4	−2.39	0.001
		Ift172	NM_026298	Mus musculus intraflagellar transport 172 homolog (Chlamydomonas)	−1.52	0.002
		Errfi1	NM_133753	Mus musculus ERBB receptor feedback inhibitor 1	−1.73	0.000
		Dmbx1	NM_130865	Mus musculus diencephalon/mesencephalon homeobox 1, transcript variant 1	1.50	0.010
		Mcoln3	AK033008	Mus musculus 12 days embryo male wolffian duct includes surrounding region cDNA	−1.68	0.008
		Ndrg4	NM_145602	Mus musculus N-myc downstream regulated gene 4	−1.55	0.009
		Frem2	NM_172862	Mus musculus Fras1 related extracellular matrix protein 2	−1.52	0.007
		Il2	NM_008366	Mus musculus interleukin 2	−1.50	0.010
Multicellular organismal development	GO:0007275	Isl1	NM_021459	Mus musculus ISL1 transcription factor, LIM/homeodomain	−1.59	0.007
		Mbp	NM_010777	Mus musculus myelin basic protein, transcript variant 7	−1.62	0.004
		Ift172	NM_026298	Mus musculus intraflagellar transport 172 homolog (Chlamydomonas)	−1.52	0.002
		Errfi1	NM_133753	Mus musculus ERBB receptor feedback inhibitor 1	1.73	0.000
		Dmbx1	NM_130865	Mus musculus diencephalon/mesencephalon homeobox 1, transcript variant 1	−1.50	0.010
		Mcoln3	AK033008	Mus musculus 12 days embryo male wolffian duct includes surrounding region cDNA	−1.68	0.008
		Ndrg4	NM_145602	Mus musculus N-myc downstream regulated gene 4	−1.55	0.009
		Frem2	NM_172862	Mus musculus Fras1 related extracellular matrix protein 2	−1.52	0.007
Cellular process	GO:0009987	Fpr-rs2	NM_008039	Mus musculus formyl peptide receptor, related sequence 2	1.51	0.005
		Il2	NM_008366	Mus musculus interleukin 2	−1.50	0.010
		Isl1	NM_021459	Mus musculus ISL1 transcription factor, LIM/homeodomain	−1.59	0.007
		Mbp	NM_010777	Mus musculus myelin basic protein, transcript variant 7	−1.62	0.004
		Slc2a4	NM_009204	Mus musculus solute carrier family 2 (facilitated glucose transporter), member 4	−2.39	0.001
		Ift172	NM_026298	Mus musculus intraflagellar transport 172 homolog (Chlamydomonas)	−1.52	0.002
		Errfi1	NM_133753	Mus musculus ERBB receptor feedback inhibitor 1	1.73	0.000
		Pcdhb10	NM_053135	Mus musculus protocadherin beta 10	−1.52	0.005
		Dmbx1	NM_130865	Mus musculus diencephalon/mesencephalon homeobox 1, transcript variant 1	−1.50	0.010
		Mcoln3	AK033008	Mus musculus 12 days embryo male wolffian duct includes surrounding region cDNA	−1.68	0.008
		C79407	NM_172578	Mus musculus expressed sequence C79407	−1.66	0.005
		Spon1	AK084717	Mus musculus 13 days embryo heart cDNA	−1.66	0.004
		Tnrc6a	AK147327	Mus musculus cDNA	−1.71	0.003
		Frem2	NM_172862	Mus musculus Fras1 related extracellular matrix protein 2	−1.52	0.007
		Olfr1355	AF042360	Mus musculus clone OR27-3 putative olfactory receptor	−1.53	0.001
		Olfr357	NM_146623	Mus musculus olfactory receptor 357	−1.52	0.008
		Olfr646	NM_147056	Mus musculus olfactory receptor 646	−1.67	0.010
System development	GO:0048731	Il2	NM_008366	Mus musculus interleukin 2	−1.50	0.010
		Isl1	NM_021459	Mus musculus ISL1 transcription factor, LIM/homeodomain	−1.59	0.007
		Mbp	NM_010777	Mus musculus myelin basic protein, transcript variant 7	−1.62	0.004
		Ift172	NM_026298	Mus musculus intraflagellar transport 172 homolog (Chlamydomonas)	−1.52	0.002
		Errfi1	NM_133753	Mus musculus ERBB receptor feedback inhibitor 1	1.73	0.000
		Dmbx1	NM_130865	Mus musculus diencephalon/mesencephalon homeobox 1, transcript variant 1	−1.50	0.010
		Mcoln3	AK033008	Mus musculus 12 days embryo male wolffian duct includes surrounding region cDNA	−1.68	0.008
		Frem2	NM_172862	Mus musculus Fras1 related extracellular matrix protein 2	−1.52	0.007
		Il2	NM_008366	Mus musculus interleukin 2	−1.50	0.010
Organ development	GO:0048513	Isl1	NM_021459	Mus musculus ISL1 transcription factor, LIM/homeodomain	−1.59	0.007
		Mbp	NM_010777	Mus musculus myelin basic protein, transcript variant 7	−1.62	0.004
		Ift172	NM_026298	Mus musculus intraflagellar transport 172 homolog (Chlamydomonas)	−1.52	0.002
		Errfi1	NM_133753	Mus musculus ERBB receptor feedback inhibitor 1	1.73	0.000
		Dmbx1	NM_130865	Mus musculus diencephalon/mesencephalon homeobox 1, transcript variant 1	−1.50	0.010
		Mcoln3	AK033008	Mus musculus 12 days embryo male wolffian duct includes surrounding region cDNA	−1.68	0.008
		Frem2	NM_172862	Mus musculus Fras1 related extracellular matrix protein 2	−1.52	0.007
Cellular developmental process	GO:0048869	Il2	NM_008366	Mus musculus interleukin 2	−1.50	0.010
		Isl1	NM_021459	Mus musculus ISL1 transcription factor, LIM/homeodomain	−1.59	0.007
		Mbp	NM_010777	Mus musculus myelin basic protein, transcript variant 7	−1.62	0.004
		Ift172	NM_026298	Mus musculus intraflagellar transport 172 homolog (Chlamydomonas)	−1.52	0.002
		Errfi1	NM_133753	Mus musculus ERBB receptor feedback inhibitor 1	1.73	0.000
		Dmbx1	NM_130865	Mus musculus diencephalon/mesencephalon homeobox 1, transcript variant 1	−1.50	0.010
		Mcoln3	AK033008	Mus musculus 12 days embryo male wolffian duct includes surrounding region cDNA	−1.68	0.008
		Frem2	NM_172862	Mus musculus Fras1 related extracellular matrix protein 2	−1.52	0.007
Anatomical structure development	GO:0048856	Il2	NM_008366	Mus musculus interleukin 2	−1.50	0.010
		Isl1	NM_021459	Mus musculus ISL1 transcription factor, LIM/homeodomain	−1.59	0.007
		Mbp	NM_010777	Mus musculus myelin basic protein, transcript variant 7	−1.62	0.004
		Ift172	NM_026298	Mus musculus intraflagellar transport 172 homolog (Chlamydomonas)	−1.52	0.002
		Errfi1	NM_133753	Mus musculus ERBB receptor feedback inhibitor 1	1.73	0.000
		Dmbx1	NM_130865	Mus musculus diencephalon/mesencephalon homeobox 1, transcript variant 1	−1.50	0.010
		Mcoln3	AK033008	Mus musculus 12 days embryo male wolffian duct includes surrounding region cDNA	−1.68	0.008
		Frem2	NM_172862	Mus musculus Fras1 related extracellular matrix protein 2	−1.52	0.007
Negative regulation of macromolecule metabolic process	GO:0010605	Il2	NM_008366	Mus musculus interleukin 2	−1.50	0.010
		Ift172	NM_026298	Mus musculus intraflagellar transport 172 homolog (Chlamydomonas)	−1.52	0.002
		Errfi1	NM_133753	Mus musculus ERBB receptor feedback inhibitor 1	1.73	0.000
		Dmbx1	NM_130865	Mus musculus diencephalon/mesencephalon homeobox 1, transcript variant 1	−1.50	0.010
		Tnrc6a	AK147327	Mus musculus cDNA	−1.71	0.003
Cell surface receptor linked signaling pathway	GO:0007166	Il2	NM_008366	Mus musculus interleukin 2	−1.50	0.010
		Ift172	NM_026298	Mus musculus intraflagellar transport 172 homolog (Chlamydomonas)	−1.52	0.002
		Plce1	AK035546	Mus musculus adult male urinary bladder cDNA	1.53	0.010
		Errfi1	NM_133753	Mus musculus ERBB receptor feedback inhibitor 1	1.73	0.000
		Olfr1355	AF042360	Mus musculus clone OR27-3 putative olfactory receptor	−1.53	0.001
		Olfr357	NM_146623	Mus musculus olfactory receptor 357	−1.52	0.008
		Olfr646	NM_147056	Mus musculus olfactory receptor 646	−1.67	0.010

A positive fold change indicates that supplementation increased the expression of the gene.

**Figure 1:**
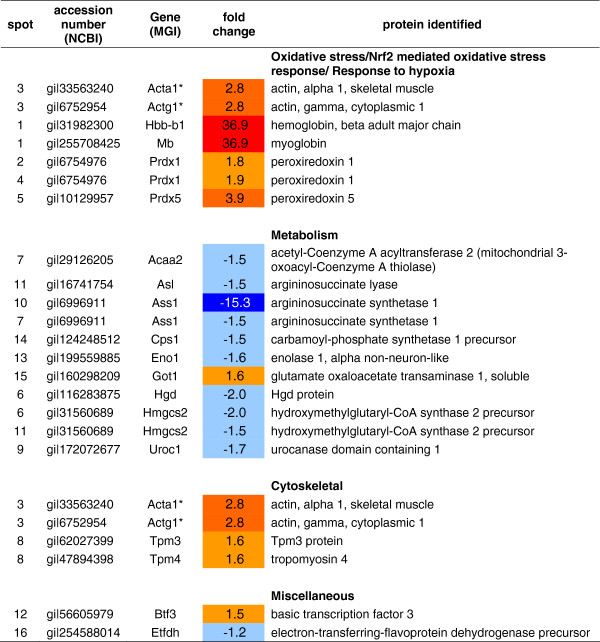
**Changes in protein expression in the liver of mice supplemented with adequate levels of selenium and folate post-weaning.** This analysis is of duplicate technical replicates representing protein pooled from the 6 mice per treatment group used for microarray analysis. A positive fold change indicates that supplementation increased the expression of the protein. Note that 16 differentially expressed protein “spot-features” were identified, representing 22 unique proteins (spot features 1, 3, 6, 7, 8, and 11 each contain 2 proteins). * Indicates that a protein appears in multiple functional groups.

Pooled differential proteomic analysis (HF-low-suf *vs.* HF-low-low) showed no effect of post-weaning supplementation with adequate levels of selenium and folate on protein expression in the colon (data not shown). In the liver, 22 proteins were differentially expressed (P < 0.05; Figure 1) and are shown in the gel image depicted in Additional file 3: Figure S1, and listed in Figure 1. These included proteins with a role in the oxidative stress response, metabolic proteins (in particular those in the urea cycle and amino acid metabolism), and cytoskeletal proteins such as actin and tropomysin (Figure 1).

**Figure 2:**
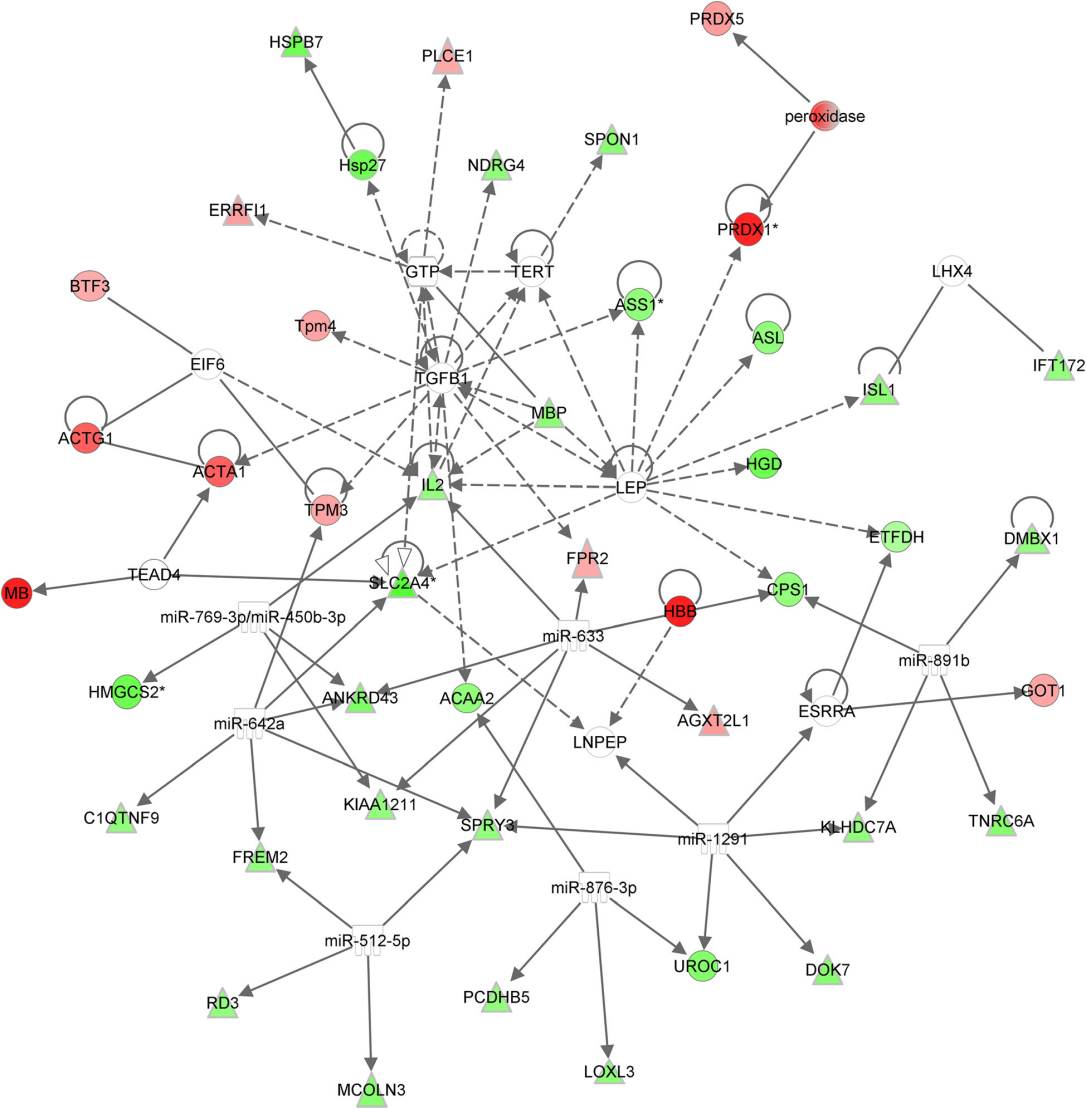
**The effects of adequate selenium and folate post-weaning on differentially expressed genes and proteins in the liver of female mice exposed to high-fat diets with low selenium and folate during gestation and lactation.** A network of genes (Δ) and proteins (Ο) was generated using Ingenuity Pathways Analysis software. All genes and proteins differentially expressed in the liver in response to altered selenium and folate levels were included, and the IPA knowledge base developed links between these based on direct interactions previously reported in the literature. Genes and gene products with higher expression in the deficient animals are shown in red, those with lower expression in green. This approach was used to identify whether any genes or proteins showed direct interactions with a large number of other genes or proteins, and may therefore be of particular importance in mediating the effects of altered dietary selenium and folate.

**Figure 3:**
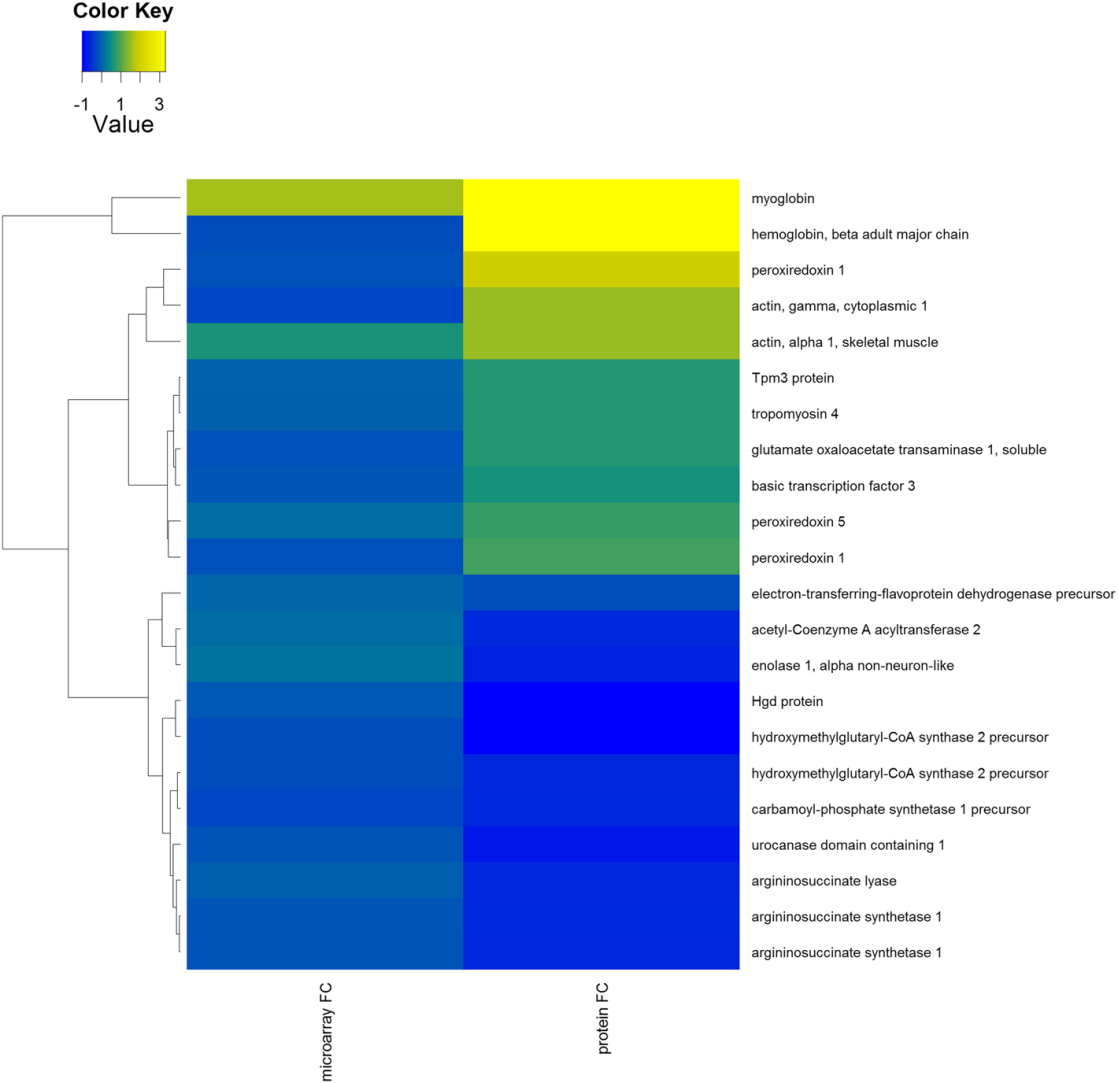
**Comparison of gene and protein data using unsupervised hierarchical clustering in the liver.** Comparison of gene (“microarray FC”) and corresponding protein (protein FC) expression levels in liver tissue of mice receiving adequate levels of folate and selenium post-weaning with expression levels in liver of mice with low dietary selenium and folate. Yellow (fold change >1) indicates greater expression in HF-Low-Suf mice, blue (fold change < 1) indicates greater expression in HF-Low-Low mice. Proteins and genes were non-hierarchically clustered according to similarity in fold change. Multiple instances of genes/protein names indicate multiple microarray probes/protein spots. Data for all gene expression changes are shown in Additional file 1: Table S1 and Additional file 2: Table S2, and for protein changes in Figure 1.

A network of interactions between genes and proteins, and the changes in expression associated with feeding of the HF-low-suf diet, can be seen in Figure 2. As shown in Figure 3, gene and protein expression fold changes in the liver were not highly correlated (Pearson Coefficient = 0.33).

### Global DNA methylation and histone modifications

There were no significant differences in the global levels of histone H3 acetylation (H3K9ac; P = 0.808) or methylation (H3K9me2; p = 0.788) detected in liver, while there was insufficient material available to accurately measure either of these changes in colon (Table 3).

**Table 3:** The effects of adequate levels of selenium and folate post-weaning life on histone H3 modifications (acetylation or methylation of lys9 residue, mean peak intensity) in the liver of mice fed high-fat diets

**Tissue**	**HF-low-low**	**HF-low-suf**		
	**n = 6**	**n = 6**	**SE**	**P-value**
H3K9ac	100.0	109.1	0.15	0.808
H3K9me2	100.0	109.1	0.14	0.788

Note that colon was not studied as insufficient protein was obtained from these tissues to perform the analysis.

### Gene-specific methylation of *Slc2a4*

**Figure 4:**
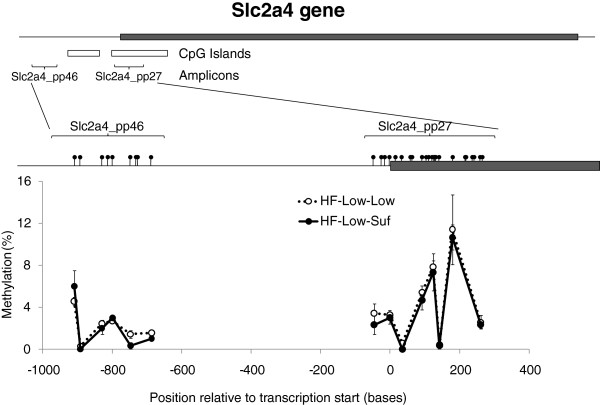
**Methylation of CpG sites at, and upstream of, the transcription start site of the *Slc2a4 *gene.** The promoter region and transcription start site of the *Slc2a4* gene were targeted to establish if differential DNA methylation was occurring at specific CpG sites within these regions. The upper panel represents the position of CpG islands, target amplicons, and CpG sites within the target region, while the graph represents mean ± SEM for the percentage methylation at each site, as measured using Sequenom EpiTYPER. No significant differences were observed as a result of selenium and folate supplementation for any of the CpG sites measured.

The expression of the *Slc2a4* gene in liver was significantly down-regulated in supplemented mice; microarray data showed consistent decreased *Slc2a4* mRNA abundance due to adequate selenium and folate supplementation, with 8 separate probes for this gene on the array showing a >2 |fold-change| (range −2.10 to −2.39). In addition, methylation of sites in the promoter region of *Slc2a4* gene has been associated with transcriptional control of this gene [25,26]. This gene was therefore chosen to investigate gene-specific methylation. Data were generated by Sequenom analysis for 6 individual CpG sites (1, 4, 6, 9, 16 and 17) and two CpG clusters (12-13-14-15, and 22–23) within the first amplicon (pp_27), and 6 individual CpG sites (1, 2, 3, 5, 6 and 9) within the second amplicon (pp_46). There were no significant differences with respect to the percentage methylation of any of these CpG sites due to selenium and folate supplementation post-weaning in mice fed high-fat diets (Figure 4), and there was also no effect on the overall pattern of methylation.

## Discussion

The study reported here shows that post-weaning supplementation with adequate levels of selenium and folate (following maternal consumption of a high-fat diet low in selenium and folate during gestation and lactation) altered metabolism-related pathways in both liver and colon. In colon, differential expression of genes was observed in DNA metabolic processes, with no changes observed in protein expression. In the liver, differential expression of both genes and proteins was observed (although not necessarily concomitant), and a reduction of total DNA methylation was also seen.

### Gene and protein expression

Analysis of the effect of post-weaning supplementation of adequate levels of selenium and folate on liver protein expression identified differential expression of proteins involved in oxidative stress response, metabolism, and cytoskeletal assembly. The changes in oxidative stress response are likely to be due to the antioxidant role of selenium being enhanced due to supplementation. Changes in expression levels of genes and their corresponding proteins were not highly correlated. This is not surprising, as the use of direct gene/protein correlation measures is unlikely to reflect the actual correspondence between the expression of a gene and its protein, which is complicated due to post-transcriptional and translational regulation [27]. Nevertheless, in this study both protein and/or gene expression patterns were altered in metabolism-related pathways (e.g., cellular process, cellular component organization, organelle organization, and DNA metabolic process pathways, multicellular organismal process, developmental process, and multicellular organismal development) indicating a potentially important developmental role for post-weaning supplementation of selenium and folate.

One gene which showed common differential expression of both transcript and protein was myoglobin, which had higher expression levels in offspring which had received the adequate diets. This is consistent with studies showing that administration of folic acid can enhance porphyrin production [28]. The haeme porphyrin ring is an essential component of both myoglobin and haemoglobin, and because folate functions as a carbon carrier in the formation of haeme [29], folate supplementation may result in increased availability of haeme. This may be one mechanism explaining the observed large increase in myoglobin gene and protein expression, and in haemoglobin protein expression, in the current study.

There were no genes that showed altered expression in both liver and colon. It is known that selenium supplementation effects selenoprotein activity differently in different tissues [30]. Furthermore, while liver is the main tissue of folate storage and metabolism [31], supplementation with methyl donors including folic acid has been shown to affect gene expression and DNA methylation in colon tissue in a mouse model of colitis [32], suggesting that colon may be an important target tissue for folate supplementation. It has also been shown that the effects of folate supplementation can differ depending on age and tissue [33]. It is therefore not surprising that there was no overlap between the pattern of gene and protein expression in these tissues in the current study, particularly given the relatively small number of genes and proteins that were differentially expressed in response to different levels of selenium and folate.

### DNA methylation

We observed a small (approximately 10%) but significant decrease of global DNA methylation in the liver of offspring fed selenium and folate supplemented. Other studies have shown varying effects of selenium and folate supplementation on global DNA methylation. For example, selenium deficiency decreased global DNA methylation in the colon, with no effect in liver, in Fischer-344 rats fed a deficient diet from weaning; in the same study, folate had no effect on global DNA methylation [34]. In contrast, and in agreement with our data, adequate (0.15 mg/kg diet) and supra-nutritional (4 mg/kg diet) selenium supplementation decreased total DNA methylation levels in the liver of rats maintained on standard diets (AIN-93 G), compared to deficient diets [35]. This study involved feeding a standard diet, whereas our study had a high-fat diet, thus the same effect of supplementation on total DNA methylation has been observed within the context of two different diets.

A reduction in overall levels of DNA methylation in the liver may indicate increased transcriptional activity, because methylation is usually inversely correlated with the transcriptional status of genes [36]. Although an overall reduction in liver DNA methylation was observed in mice supplemented with selenium and folate in the current study, genes differentially expressed in the liver largely showed reduced expression in response to supplementation. It has recently been observed that the epigenetic effects of folate on DNA methylation are highly complex, with factors such as gene and site specificity, cell type, target organ, degree and duration of folate manipulations, and interactions with other methyl group donors and dietary factors all potentially influencing the effects [37]. It is therefore perhaps not surprising that there is not a simple link between increased dietary folate and the degree of methylation, nor between methylation and gene expression. Results from our study may also be complicated by potential interactions between folate and selenium. Furthermore, a recently reported human population study found no positive associations between intake of methyl donor nutrients during pregnancy and overall DNA methylation in offspring [38].

Folate depletion has been associated with gene-specific changes in DNA methylation, for example of the *p53*[39] and *Slc394a*[40] genes. Information on concurrent selenium and folate supplementation is less widely available. In the current study, we observed decreased expression of the *Slc2a4* gene (which encodes the glucose transporter GLUT4) in offspring of the adequate selenium/folate group. It has been observed that combining selenium with insulin was able to restore, among other things, disturbances in GLUT4 levels in cardiac muscle in diabetic rats. Furthermore, methylation of sites in the promoter region of *Slc2a4* gene has been associated with transcriptional control of this gene [25,26]. In this study, however, we found no association between changes in *Slc2a4* expression and altered methylation in the promoter region. The site of methylation may be an important factor in the regulation of gene expression, with methylation of CpG sites in the promoter region closest to the transcription start site having more influence on gene expression than those further from this region due to the effects of methylation on transcription factor binding [41]. Several studies have also shown that CpG methylation within introns is associated with changes in gene expression [42-44]. It is therefore possible that CpG sites within the *Slc2a4* gene that were not analysed in the current study were differentially methylated, and thereby influenced its levels of expression. It is also plausible that the overall reduction in methylation observed in this study may have occurred in non-coding regions of the genome, or that a ‘redistribution’ of methyl marks may have occurred in response to altered dietary folate/selenium. A more comprehensive analysis of methylation, both across the *Slc2a4* gene and at a genome-wide level, would be required to further clarify these points.

It is also important to note that CpG methylation is only one possible factor by which gene expression may be regulated [41]. For example, modifications of chromatin structure, including acetylated histone H3, acetylated histone H4 and di-methylated histone H3 at lysine 4, within the promoter region have been shown to regulate expression of the *Slc2a4* gene [45]. Additional studies would be required to clarify any role of these modifications in the observed change in expression of this gene.

### Histone modifications

Previous research has shown that in addition to causing promoter DNA demethylation, selenium (in the form of selenite) can decrease histone deacetylase activity, increase levels of acetylated lysine 9 on histone H3 (H3-Lys 9), and (as a result of the increased acetylation) decrease levels of methylated H3-Lys 9 in prostate cancer cell lines [46]. Supplementation with the methyl group donors folate (2 mg/kg), methionine (0.4%), and choline bitartrate (0.3%) has been shown to affect histone modifications (decreasing histone H3K9me3 levels) when compared with mice fed a diet with low folate, and lacking choline and methionine [47]. Furthermore, studies of a choline and methyl-deficient C57BL/6 mouse prostate cancer model suggest that chromatin modifications are more susceptible to methyl-deficient diets than DNA methylation at certain loci [48].

The current study is the first to our knowledge that has attempted to investigate the effects of a combination of selenium and folate on histone modifications. We showed adequate selenium and folate post-weaning did not affect global histone modifications in the liver of mice fed high-fat diets. There could be a number of reasons for this. Previous studies have used diets deficient in other methyl donors such as choline and methionine. This suggests that folate deficiency, even in the presence of a selenium deficiency, may not be sufficient to trigger global histone changes in the tissues studied. In addition, it may be that there are modifications occurring at histone sites associated with specific genes, and the global approach we have applied was not sufficiently sensitive to evaluate such changes. Future studies including measurement of gene-specific histone modifications using, for example, chromatin immunoprecipitation (ChIP) experiments would help to clarify this point.

## Conclusions

The data described here show that in addition to changes in gene and protein expression in metabolic and oxidative pathways, altering dietary levels of selenium and folate post-weaning can alter global DNA methylation. Because epigenetic mechanisms such as DNA methylation may have a role in disease processes, modulation of these by micronutrient supplementation may play a role in preventing or ameliorating negative health outcomes from inadequate nutrition. Further research is required to establish if epigenetic mechanisms are mediating the specific changes observed in gene and protein expression, and if so which are the precise mechanisms involved. In addition, further studies are required to establish whether there are any confounding effects due to combining selenium and folate, or to establish whether there is any interaction between these two compounds.

Finally, it has been suggested that a five week dietary ‘run-in’ period is required to systemically reduce folate levels in mice [49], and data from rats suggest that at least 4 weeks may be required to deplete selenium [50]. While liver selenium content of the HF-low-low animals was less than that of the HF-low-suf animals in our study, it was not as low as has been observed in other studies which used a similar level of dietary selenium but had a longer feeding period [51]. While we do not have data on liver or colon folate levels, or colon selenium levels, of the mice in our study due to insufficient sample being available, it seems possible that these levels would not have been significantly reduced until early lactation due to our relatively short run-in period. Future studies could include a minimum five week run-in period, as well as measurement of selenium and folate [52] levels in red blood cells, colon and liver, to confirm that significant depletion had occurred and to more clearly link this depletion with the outcome measures of the study.

## Methods

### Animals & diets

All procedures involving animals were approved by the Grasslands Animal Ethics Committee under the New Zealand Animal Welfare Act 1999.

A pelleted “Western” diet [53,54] (D12079B; Research Diets, New Brunswick, NJ 08901, USA) containing 40%, 42% and 17% kcal from fat, carbohydrate and protein, respectively (Additional file 4: Table S3) was modified to contain either 0.4 mg/kg folate and 0.1 mg/kg selenium (high-fat, low selenium and folate; HF-low) or 1.8 mg/kg folate and 0.60 mg/kg selenium (high-fat, adequate selenium and folate; HF-suf). Analysis of the diets after manufacture confirmed that these were the actual levels of selenium and folate. These levels of folate and selenium are commonly used for supplementation in mouse and rat diets [34,55,56] and are also in accordance with National Research Council guidelines for Laboratory Animals. Additional ethoxyquin was included in both diets to prevent rancidity (Additional file 4: Table S3).

Thirty five female wild type C57BL-6 mice (Animal Resource Centre, Western Australia) were fed a HF-Low diet for 7 days prior to mating with male C57BL-6 mice (Ruakura Small Animal Facility, Hamilton, New Zealand). An important epigenetic period is oocyte maturation, which in the mouse is 3.5 days prior to fertilisation [57]. The experimental diet was therefore introduced prior to the phase of oestrus during which pregnancy occurs, i.e., a minimum of 4 days prior to mating [58]. Once pregnancy was confirmed via the presence of a vaginal plug, males were removed from the mating cages. The breeding dams were maintained on the HF-low diets during mating, gestation and lactation. Offspring remained with their dams until weaning (*c*. 28 days of age). At weaning, female offspring were randomly allocated to either the HF-low diet (n = 6; HF-low/low) or switched to the HF-Suf diet (n = 6; HF-low/suf) until 12 weeks of age. Mice were offered 20 g of food pellets twice weekly and had *ad libitum* access to water. Food intake (estimated by collecting and weighing uneaten food) and bodyweight were determined twice weekly. General health score was determined daily.

The mice were euthanised at 12 weeks of age via CO_2_ asphyxiation and cervical dislocation. Prior to tissue collection, mice were fasted and re-fed to minimise variation in food intake immediately before sampling [59]. Colon tissue and liver were excised, washed in cold saline and stored in tubes containing 1 mL of RNA later, then stored at 4°C overnight. The RNA later was removed and the tissues snap-frozen in liquid nitrogen and stored at −85°C until analysis.

### RNA and DNA isolation

Genomic DNA, total RNA and protein from whole colon and liver tissue was extracted using an AllPrep® DNA/RNA/Protein mini kit (Qiagen, Cat number 80004). DNA was quantified using a NanoDrop ND1000 (Thermo Fisher Scientific) and stored at −20°C until required for further analyses. Quality of the genomic DNA for subsequent analyses was determined by agarose gel electrophoresis. Total RNA was quantified using a NanoDrop ND1000 and RNA quality was determined using an Agilent 2100 Bioanalyser (Agilent Technologies, Palo Alto, CA, USA) to measure the RNA integrity number (RIN). RNA was stored at −85°C until required for microarray analysis.

### Microarray hybridization and analysis

Microarray hybridization has been described in detail elsewhere [60]. Briefly, a reference design was used for microarray hybridization: intestinal and liver RNA extracts from all mice were pooled in an equimolar proportion and used as the reference sample. Cy3-labelled sample cRNA (0.75 μg) and Cy5-labelled reference cRNA (0.75 μg) was prepared using an *in situ* hybridization kit-plus (Agilent Technologies Inc., Palo Alto, California, USA) and hybridised to Agilent Technologies Mouse G4122F – 4x44k 60mer oligonucleotide arrays, as previously described [61]. Slides were scanned using an Agilent scanner after an automatic gain and calibration prior to each scan. Spot identification and quantification were performed using Agilent Feature Extraction software version 9.5. Microarray fluorescence signals were normalized using a global loess algorithm in R2.14.1 with the Linear Models for Microarray Data (limma) package [62]. Differentially expressed genes were determined using an Empirical Bayes modified t-statistic from a linear model of microarray analysis. Genes with a greater than 1.5 fold change and a P value less than 0.01 were considered to be differentially expressed. Significantly over-represented Gene Ontology biological processes among differentially expressed genes were determined using the GOStats package in R 2.14.1 [63]. The data discussed in this publication have been deposited in NCBI’s Gene Expression Omnibus [64] and are accessible through GEO Series accession number GSE43038 (http://www.ncbi.nlm.nih.gov/geo/query/acc.cgi?acc = GSE43038).

### Protein expression profiles and analysis

Sample preparation, 2D-gel electrophoresis, LC-MS peptide analysis and database searching were all performed as previously described [65]. Briefly, duplicate technical replicates of pooled aliquots of protein (50 μg per treatment, representing protein pooled from the 6 mice per treatment group used for microarray analysis) were labeled with 200 pmol CyDyes (GE Healthcare) as per manufacturer’s instructions. In all cases, HF-low-low mice were labeled with Cy2 and HF-low-suf samples were labeled with Cy5. Each control and test pair was mixed to give 100 μg of protein and run on the same first dimension strip, which (along with equilibration, second dimension, scanning, and staining) was performed as previously described [65]. Spot detection, warping, and statistical analysis were performed using Delta2D 4.2 software from Decodon (Greifswald, Germany). For accurate spot detection, settings were chosen according to manufacturer’s instructions (Decodon). Gel images were first warped using a combination of the ‘exact’ warping setting with additional manual vector adjustment. A fusion gel image was created using the ‘union’ mode (a weighted mean across the gel series). Spot detection on the fusion gel was performed automatically by the software, and manual editing was used to create the end product, spot boundaries from which were then transferred to all gels in the series. Normalised values following background subtraction were calculated by the software and a ratio of normalised volumes to that of the parent gel was used for quantitative analysis of protein expression level.

Differentially expressed proteins were only flagged as significant where the fold abundance was greater than 2, and the *t*-test value was greater than 95%. Digestion of the differentially expressed proteins for subsequent identification was carried out as previously described [65].

Tryptic peptides were separated and analyzed using an Ettan multidimensional liquid chromatography system (GE Healthcare) coupled to an LTQ linear ion trap mass spectrometer with a nano-spray ionization interface (ThermoQuest, Finnigan, San Jose, CA, USA) [66]. MS/MS data were analyzed using TurboSEQUEST protein identification software [67,68] and spectra were searched against the National Center for Biotechnology Information (NCBI) *Mus musculus* database. All matched peptides were confirmed by visual examination of the spectra.

### Functional analysis of proteins

Functional classification of proteins was based on GeneOntology using the Mouse Genome Informatics (MGI) database (http://www.informatics.jax.org/), the Kyoto Encyclopedia of Genes and Genomes (KEGG) encyclopedia (http://www.genome.jp/kegg/genes.html), and Ingenuity Pathways Analysis (IPA) (Ingenuity Systems, http://www.ingenuity.com/), as previously described [66].

### Global DNA methylation and histone modifications

Genomic DNA extracted from the colon and liver tissues was used to determine global DNA methylation using high performance liquid chromatography (HPLC) as described previously [69]. Briefly, 50 μg genomic DNA was treated with RNase enzymes to remove contaminating RNA, then enzymatically hydrolysed to mononucleotides. The samples were run on the HPLC and detected at an absorbance of 280 nm. The relative amounts of methylated and unmethylated cytosine were determined using the area under the peak for each nucleotide.

In order to assess the effects of adequate levels of selenium and folate supplementation on histone modifications profiles in the liver previously reported methods [70,71] were modified in order to significantly increase the concentration of nuclear proteins by combining both the nuclear pellet and supernatant. 50 mg of frozen tissue was homogenised in 0.5 ml of ice-cold low salt extraction buffer (10 mM HEPES (pH 7.9), 10 mM KCl, 1.5 mM MgCl_2,_ 1 mM DTT, 5 mM sodium butyrate, 20 mM NaF and a complete protease inhibitor cocktail (Roche)) and incubated on ice for 15 min. Igepal was added to a final concentration of 0.1% and incubated for a further 20 min on ice. Pellets remaining after centrifugation (17 500 × *g*, 5 min, 4°C) were washed four times with low salt extraction buffer then resuspended in 150 μl of ice-cold high salt extraction buffer (920 mM HEPES (pH 7.9), 420 mM NaCl, 1.5 mM MgCl_2,_ 0.2 mM EDTA, 5 mM sodium butyrate, 1 mM DTT, 20 mM NaF and a complete protease inhibitor cocktail). Samples were incubated on ice for 30 min using a rocking platform and centrifuged at 17 500 × *g*, 4°C for 15 min. The supernatant was collected and stored on ice while the pellet was resuspended in a minimal volume of precipitation buffer (50 mM Tris–HCl (pH 7.6), 10 mM NaCl, 2 mM EDTA, 1 mM PMSF, 10 μg/ml aprotinin, 1% Igepal, 5 mM sodium butyrate and DNAse to a final concentration of 5 μg/mL), incubated for 30 minutes at room temperature to degrade genomic DNA, and centrifuged (17 500 × *g*, 4°C, 1 min). This supernatant was added to the previous supernatant and stored at −80°C for further studies. Protein concentration was determined using the Thermo Scientific Pierce Coomassie (Bradford) Protein Assay Kit according to the manufacturer’s instructions (Thermofisher Scientific Inc., Rockford, Illinois, USA).

Western blot analysis was performed in triplicate on nuclear protein extracts (20 μg) isolated from liver using polyclonal anti-acetylated histone 3 (lys9 (H3K9ac); Cell Signaling Technology, Inc., Danvers, Massachusetts, USA), and polyclonal anti-dimethylated histone 3 (lys9 (H3K9me2); Cell Signaling) antibodies. Equal loading of samples was confirmed by staining with coomassie blue (Simply Blue™ Safestain, Invitrogen, Carlsbad, California, USA). Films were scanned and bands analysed using the ImageJ software to determine the average peak density. Statistical analysis of results was performed using a two tailed Student’s *t*-Test. Correlation analysis between values for the two types of histone modification was performed using the Spearman rho correlation coefficient.

### Gene specific DNA methylation

Microarray data showed consistent increased *Slc22a4* mRNA abundance due to selenium and folate supplementation, with 8 separate probes for this gene on the array showing a >2-fold down-regulation (range −2.10 to −2.39) therefore this gene was chosen to investigate the effects of post-weaning selenium and folate supplement on gene-specific DNA methylation in mice fed high-fat diets.

Genomic DNA was purified from liver tissue using the AllPrep® DNA/RNA/Protein mini kit as described above. Quantitative analysis of DNA methylation of *Slc22a4* was performed using the Sequenom MassARRAY Compact System (http://www.sequenom.com/). Briefly, this involves the gene-specific amplification of bisulfite-treated DNA, followed by *in vitro* transcription and analysis by matrix-assisted laser desorption ionization time-of-flight (MALDI-TOF) mass spectrometry [72]. 1 μg of DNA was bisulfite-converted using an EZ DNA Methylation kit (Zymo Research, Irvine, California, USA). Sequenom EpiDesigner software was used to design PCR primers specific for bisulfite-converted DNA. Each reverse primer contained a T7-promoter tag for *in vitro* transcription (5^′^-cagtaatacgactcactatagggagaaggct-3^′^), and the forward primer was tagged with a 10mer to balance Tm (5^′^-aggaagagag-3^′^). Two sets of primer pairs were designed, generating two amplicons: one of 374 bases (referred to as “pp_27”) encompassing the transcription start site of the *Slc22a4* gene and including part of a CpG island within that region, and a second of 323 bases (“pp_46”) 976 base pairs upstream of the transcription start site and immediately upstream of a CpG island in that region. Details of the primer pairs are shown in Table 4, and their positions relative to the *Slc22a4* transcription start site are shown in Figure 4.

**Table 4:** Primers for sequenom analysis of CpG methylation of the Slc2a4 promoter region

**Amplicon ID**	**mus_Slc2a4_pp27**	**mus_Slc2a4_pp46**
**Genomic co-ordinates**	chr11:69761390-69761763	chr11:69762346-69762668
**Strand**	–	–
**Length**	374	323
**Sequence**	CCTGGCCAATGGGTGTT	
	GTGAAGGGCGTGTCCTA	GGGAAGGGTTAATAGAA
	TGGCGGGGCGGGAGTGG	GAGAGCCACCCCAGAA
	GGAGGTGGCTTCAGCTCT	GTTAGCTACCCTGGTGC
	CCGCATCTTTCCCCCTCA	AATCCACTAAGGTTCCT
	AGCGGGTCTCACTAGAT	CGCTCTCCCTCTAGGTG
	CCCGGAGAGCCTTGGTG	GCGCCAGAAGCCTTGC
	CTCTCCGGTTCCGTGGGT	ACTTCTCTTGGGCCTTTT
	TGTGGCAGTGAGTCCCAC	TCTGAATTGAGCTCTCTC
	CAGACCCGCCCTTTGCAC	TCCACATTCTTCGCCAGC
	ACGGCTTCCGAACGCCGG	TCTCTCCCTGGACGTGCTT
	GGTCTCGTGCCGGCCAG	AGGTCGTGCCCTCTCAGCT
	GCCCGGACCCTATACCC	GTAGACCCAAAACAGTAG
	TATTCATTTTTTTCTTAT	CTGACTCTGGAAAGCTTGT
	TGCAGCGCCTGAGTCTT	CGCCCACGCGGCCAGCACA
	TTCTTCTTTTAAAACAAG	TGCCTGGAGGCTCAGGGA
	ATGCCGTCGGGTTTCCAG	CTTCAGGGAGGGTGGTGT
	CAGATCGGCTCTGACGTAA	GACTGGCGTGAGCACCTG
	GGTTCAGCATACCGGGGC	TCCCTTGGGTCCCCTCCAAGA
	GAATTGGGAAATCTGGTC	
	CAGTTTTTCTTGGGCTGAGTT	
**Left primer length**	23	27
**Left primer sequence**	aggaagagagTTTGGTTAAT	aggaagagagGGGAAGGGTTA
	GGGTGTTGTGAAG	ATAGAAGAGAGTTATT
**Right primer length**	25	25
**Right primer sequence**	cagtaatacgactcactatagggagaag	cagtaatacgactcactatagggagaagg
	gctAACTCAACCCAAAAAA	ctTCTTAAAAAAAACCCAA
	AACTAAACC	AAAACAAA

Bisulfite-treated DNA was PCR amplified (Qiagen HotStar Taq Polymerase; Bio-Strategy Ltd, Auckland, NZ) and treated with Shrimp Alkaline Phosphatase (Sequenom, Queensland, Australia), heat inactivated, and a simultaneous *in vitro* transcription/uracil-cleavage reaction was carried out. Transcription cleavage products were desalted and spotted on a 384-pad SpectroCHIP (Sequenom) using a MassARRAY nanodispenser (Samsung). Mass spectra were acquired using a MassARRAY MALDI-TOF MS (Bruker-Sequenom) and peak detection, signal-to-noise calculations and quantitative CpG site methylation analysis were performed using proprietary EpiTyper software v1.0.5 (Sequenom). For fragments containing a single CpG site, DNA methylation state was calculated by the ratio of methylated to un-methylated fragments. For cleavage products containing multiple CpG sites the average methylation status of the fragment is reported.

### Analysis of liver selenium

Liver selenium content was measured using HNO_3_/HClO_4_ digestion, followed by hydride generation atomic absorption spectroscopy. Analysis was performed by Eurofins New Zealand (Ruakura Research Centre, Hamilton, New Zealand; http://www.eurofins.co.nz/).

### Statistical analysis

Food intake and bodyweight data were analysed using the Residual Maximum Likelihood (REML) method with a Repeated Measurements Model fitted (GenStat Release 12.2). Global DNA methylation data and liver selenium data were compared using analysis of variance (ANOVA; GenStat Version 9). Gene-specific methylation data was analysed using REML (GenStat Release 14.1). Additional statistical analyses were performed as described for the specific method in question. All data relate to n = 6 mice per treatment. This is because data from our previous studies [73,74] have demonstrated that microarray analysis of RNA obtained from either 5 or 6 individual mice per treatment is sufficient to robustly identify differentially expressed transcripts. We therefore used RNA from 6 animals in each treatment group for microarray analysis in the current study, and used data derived from these mice for all other analyses.

## Competing interests

The authors declare that they have no competing interests.

## Authors’ contributions

ENB conducted the research. Laboratory and/or data analysis was conducted by: SAB (histone modifications), ENB and WY (gene expression profiles), JMC, DTB, WAL (protein expression profiles), gene-specific DNA methylation (MPGB) ENB, MPGB, WCM and NCR designed the study. ENB, SB, MPGB, JMC, NCR and WY contributed to the writing of this paper. All authors read and approved the final manuscript.

## Authors’ information

Emma N Bermingham, Shalome A Bassett, Wayne Young, Nicole C Roy, Warren C McNabb, Janine M Cooney, Di T Brewster, William A Laing and Matthew PG Barnett: Nutrigenomics, New Zealand (http://www.nutrigenomics.org.nz/).

## Pre-publication history

The pre-publication history for this paper can be accessed here:

http://www.biomedcentral.com/1755-8794/6/7/prepub

## Supplementary Material

Additional file 1: Table S1 Differentially expressed genes in the colon of mice supplemented with adequate levels of selenium and folate post-weaning (n = 6 per treatment). A positive fold change indicates that supplementation increased the expression of the gene. Click here for file

Additional file 2: Table S2 Differentially expressed genes in the liver of mice supplemented with adequate levels of selenium and folate post-weaning (n = 6 per treatment). A positive fold change indicates that supplementation increased the expression of the gene. Click here for file

Additional file 3: Figure S1 2D-DIGE gel representing differentially expressed proteins identified in the liver tissue of female C57 mouse fed a high-fat diet supplemented with adequate selenium and folate (HF-low-suf) born to mothers fed a high-fat diet without supplementation, compared to female offspring maintained on the un-supplemented diet (HF-low-low). Protein annotations are shown in Figure 1 of the main text. The approximate pI and molecular weight (MW) in kDa are given on the x and y axes, respectively. Click here for file

Additional file 4: Table S3 Composition of diets used to determine the effects of post-weaning supplementation with adequate levels of selenium and folate in mice fed high-fat diets. Click here for file
