# Supplementary material for: Post-weaning selenium and folate supplementation affects gene and protein expression and global DNA methylation in mice fed high-fat diets
Source: BMC Med Genomics. 2013 Mar 5;6:7. doi: 10.1186/1755-8794-6-7 (PMC3599545; doi:10.1186/1755-8794-6-7)
Supplement: Additional file 1: Table S1 — Differentially expressed genes in the colon of mice supplemented with adequate levels of selenium and folate post-weaning (n = 6 per treatment). A positive fold change indicates that supplementation increased the expression of the gene. [file 1755-8794-6-7-S1.doc]

## Additional file 1. Differentially expressed genes in the colon of mice supplemented with adequate levels of selenium and folate post-weaning (n=6 per treatment). A positive fold change indicates that supplementation increased the expression of the gene.

| **Probe Name** | **Gene Name** | **Systematic Name** | **Description** | **log FC** | **Fold Change** | **P Value** |
| --- | --- | --- | --- | --- | --- | --- |
| A_51_P460391 | 2010016B13Rik | NM_183253 | Mus musculus RIKEN cDNA 2010016B13 gene (2010016B13Rik), mRNA [NM_183253] | -1.24 | 2.36 | 0.006 |
| A_52_P1187697 | AK039199 | AK039199 | Mus musculus adult male hypothalamus cDNA, RIKEN full-length enriched library, clone:A230106P21 product:unclassifiable, full insert sequence. [AK039199] | -0.59 | -1.51 | 0.009 |
| A_52_P843919 | AK086179 | AK086179 | Mus musculus 15 days embryo head cDNA, RIKEN full-length enriched library, clone:D930010N06 product:unclassifiable, full insert sequence. [AK086179] | -0.75 | -1.68 | 0.006 |
| A_51_P274274 | Arid4a | AK087354 | Mus musculus 0 day neonate lung cDNA, RIKEN full-length enriched library, clone:E030049C01 product:unclassifiable, full insert sequence. [AK087354] | -0.64 | -1.56 | 0.006 |
| A_52_P102413 | AW549877 | NM_145930 | Mus musculus expressed sequence AW549877 (AW549877), mRNA [NM_145930] | -0.62 | -1.54 | 0.009 |
| A_52_P502849 | BC108385 | BC108385 | Mus musculus cDNA clone MGC:118186 IMAGE:4989625, complete cds. [BC108385] | -0.59 | -1.51 | 0.010 |
| A_52_P115638 | Col4a3bp | NM_023420 | Mus musculus procollagen, type IV, alpha 3 (Goodpasture antigen) binding protein (Col4a3bp), mRNA [NM_023420] | -0.62 | -1.54 | 0.004 |
| A_51_P494446 | D4Ertd429e | NM_001025106 | Mus musculus DNA segment, Chr 4, ERATO Doi 429, expressed (D4Ertd429e), transcript variant 1, mRNA [NM_001025106] | -0.66 | -1.58 | 0.010 |
| A_52_P421918 | Hel308 | BC082601 | Mus musculus helicase, mus308-like (Drosophila), mRNA (cDNA clone MGC:105224 IMAGE:30662103), complete cds. [BC082601] | -0.65 | -1.57 | 0.008 |
| A_52_P498219 | Heph | NM_181273 | Mus musculus hephaestin (Heph), transcript variant 2, mRNA [NM_181273] | -0.61 | -1.53 | 0.001 |
| A_52_P374157 | Kbtbd8 | NM_001008785 | Mus musculus kelch repeat and BTB (POZ) domain containing 8 (Kbtbd8), mRNA [NM_001008785] | -0.63 | -1.55 | 0.009 |
| A_52_P290579 | Mef2a | NM_001033713 | Mus musculus myocyte enhancer factor 2A (Mef2a), mRNA [NM_001033713] | -0.71 | -1.64 | 0.001 |
| A_52_P140394 | Mtmr1 | NM_016985 | Mus musculus myotubularin related protein 1 (Mtmr1), mRNA [NM_016985] | -0.69 | -1.61 | 0.008 |
| A_52_P313656 | NAP123523-1 | NAP123523-1 | Unknown | -0.70 | -1.62 | 0.007 |
| A_52_P105537 | Nov | NM_010930 | Mus musculus nephroblastoma overexpressed gene (Nov), mRNA [NM_010930] | -0.78 | -1.72 | 0.005 |
| A_52_P8100 | Plscr3 | NM_023564 | Mus musculus phospholipid scramblase 3 (Plscr3), mRNA [NM_023564] | -0.65 | -1.57 | 0.007 |
| A_51_P189405 | Podxl2 | NM_176973 | Mus musculus podocalyxin-like 2 (Podxl2), mRNA [NM_176973] | -0.67 | -1.59 | 0.005 |
| A_51_P191393 | Pom121 | NM_148932 | Mus musculus nuclear pore membrane protein 121 (Pom121), mRNA [NM_148932] | -0.75 | -1.68 | 0.004 |
| A_52_P652950 | Rora | AK087905 | Mus musculus 2 days pregnant adult female ovary cDNA, RIKEN full-length enriched library, clone:E330039D10 product:unclassifiable, full insert sequence [AK087905] | -0.85 | -1.80 | 0.001 |
| A_52_P209748 | Shprh | AK037266 | Mus musculus 16 days neonate thymus cDNA, RIKEN full-length enriched library, clone:A130002D10 product:unclassifiable, full insert sequence. [AK037266] | -0.64 | -1.56 | 0.003 |
| A_51_P381584 | Stx6 | NM_021433 | Mus musculus syntaxin 6 (Stx6), mRNA [NM_021433] | -0.64 | -1.56 | 0.003 |
| A_51_P379905 | TC1494028 | TC1494028 | Q9Z263 (Q9Z263) Major sperm fibrous sheath protein Pro-mAKAP82, complete [TC1494028] | -0.62 | -1.54 | 0.008 |
| A_52_P415299 | Top2a | NM_011623 | Mus musculus topoisomerase (DNA) II alpha (Top2a), mRNA [NM_011623] | -0.80 | -1.74 | 0.006 |
