# Supplementary material for: Post-weaning selenium and folate supplementation affects gene and protein expression and global DNA methylation in mice fed high-fat diets
Source: BMC Med Genomics. 2013 Mar 5;6:7. doi: 10.1186/1755-8794-6-7 (PMC3599545; doi:10.1186/1755-8794-6-7)
Supplement: Additional file 2: Table S2 — Differentially expressed genes in the liver of mice supplemented with adequate levels of selenium and folate post-weaning (n = 6 per treatment). A positive fold change indicates that supplementation increased the expression of the gene. [file 1755-8794-6-7-S2.doc]

## Additional file 2. Differentially expressed genes in the liver of mice supplemented with adequate levels of selenium and folate post-weaning (n=6 per treatment). A positive fold change indicates that supplementation increased the expression of the gene.

| **Probe Name** | **Gene Name** | **Systematic Name** | **Description** | **log FC** | **Fold Change** | **P Value** |
| --- | --- | --- | --- | --- | --- | --- |
| A_52_P531610 | Agxt2l1 | AK030395 | Mus musculus adult male pituitary gland cDNA, RIKEN full-length enriched library, clone:5330407B06 product:weakly similar to ALANINE:GLYOXYLATE AMINOTRANSFERASE 2 HOMOLOG 1, SPLICE FORM 1 [Homo sapiens], full insert sequence [AK030395] | 0.83 | 1.78 | 0.008 |
| A_51_P382393 | Errfi1 | NM_133753 | Mus musculus ERBB receptor feedback inhibitor 1 (Errfi1), mRNA [NM_133753] | 0.79 | 1.73 | 0.000 |
| A_52_P19519 | Plce1 | AK035546 | Mus musculus adult male urinary bladder cDNA, RIKEN full-length enriched library, clone:9530064F18 product:PANCREAS-ENRICHED PHOSPHOLIPASE C homolog [Homo sapiens], full insert sequence. [AK035546] | 0.61 | 1.53 | 0.010 |
| A_51_P288138 | Fpr-rs2 | NM_008039 | Mus musculus formyl peptide receptor, related sequence 2 (Fpr-rs2), mRNA [NM_008039] | 0.59 | 1.51 | 0.005 |
| A_52_P654534 | Dmbx1 | NM_130865 | Mus musculus diencephalon/mesencephalon homeobox 1 (Dmbx1), transcript variant 1, mRNA [NM_130865] | -0.59 | -1.50 | 0.010 |
| A_52_P541353 | Il2 | NM_008366 | Mus musculus interleukin 2 (Il2), mRNA [NM_008366] | -0.59 | -1.50 | 0.010 |
| A_52_P20639 | 3322402L07Rik | NM_023727 | Mus musculus RIKEN cDNA 3322402L07 gene (3322402L07Rik), mRNA [NM_023727] | -0.59 | -1.51 | 0.007 |
| A_51_P476127 | Spry3 | NM_001030293 | Mus musculus sprouty homolog 3 (Drosophila) (Spry3), mRNA [NM_001030293] | -0.59 | -1.51 | 0.004 |
| A_51_P283423 | Ift172 | NM_026298 | Mus musculus intraflagellar transport 172 homolog (Chlamydomonas) (Ift172), mRNA [NM_026298] | -0.60 | -1.52 | 0.002 |
| A_51_P214503 | Frem2 | NM_172862 | Mus musculus Fras1 related extracellular matrix protein 2 (Frem2), mRNA [NM_172862] | -0.60 | -1.52 | 0.007 |
| A_51_P340525 | Olfr357 | NM_146623 | Mus musculus olfactory receptor 357 (Olfr357), mRNA [NM_146623] | -0.60 | -1.52 | 0.008 |
| A_51_P106000 | C530008M17Rik | AK122469 | Mus musculus mRNA for mKIAA1211 protein. [AK122469] | -0.60 | -1.52 | 0.008 |
| A_51_P289066 | Pcdhb10 | NM_053135 | Mus musculus protocadherin beta 10 (Pcdhb10), mRNA [NM_053135] | -0.60 | -1.52 | 0.005 |
| A_51_P117015 | Olfr1355 | AF042360 | Mus musculus clone OR27-3 putative olfactory receptor mRNA, partial cds. [AF042360] | -0.62 | -1.53 | 0.001 |
| A_52_P628369 | Klhdc7a | NM_173427 | Mus musculus kelch domain containing 7A (Klhdc7a), mRNA [NM_173427] | -0.62 | -1.54 | 0.008 |
| A_52_P200610 | C1qtnf9 | NM_183175 | Mus musculus C1q and tumor necrosis factor related protein 9 (C1qtnf9), mRNA [NM_183175] | -0.63 | -1.55 | 0.004 |
| A_51_P500135 | Ndrg4 | NM_145602 | Mus musculus N-myc downstream regulated gene 4 (Ndrg4), mRNA [NM_145602] | -0.63 | -1.55 | 0.009 |
| A_52_P274273 | Cdc37l1 | AK048044 | Mus musculus 16 days embryo head cDNA, RIKEN full-length enriched library, clone:C130031H07 product:cell division cycle 37 homolog (S. cerevisiae)-like, full insert sequence. [AK048044] | -0.64 | -1.56 | 0.002 |
| A_52_P554536 | Tnfrsf26 | NM_175649 | Mus musculus tumor necrosis factor receptor superfamily, member 26 (Tnfrsf26), mRNA [NM_175649] | -0.65 | -1.57 | 0.003 |
| A_52_P219233 | Loxl3 | NM_013586 | Mus musculus lysyl oxidase-like 3 (Loxl3), mRNA [NM_013586] | -0.65 | -1.57 | 0.002 |
| A_52_P337246 | Isl1 | NM_021459 | Mus musculus ISL1 transcription factor, LIM/homeodomain (Isl1), mRNA [NM_021459] | -0.67 | -1.59 | 0.007 |
| A_51_P349205 | Mbp | NM_010777 | Mus musculus myelin basic protein (Mbp), transcript variant 7, mRNA [NM_010777] | -0.69 | -1.62 | 0.004 |
| A_52_P242321 | Spon1 | AK084717 | Mus musculus 13 days embryo heart cDNA, RIKEN full-length enriched library, clone:D330035F22 product:unclassifiable, full insert sequence. [AK084717] | -0.73 | -1.66 | 0.004 |
| A_52_P148212 | C79407 | NM_172578 | Mus musculus expressed sequence C79407 (C79407), mRNA [NM_172578] | -0.73 | -1.66 | 0.005 |
| A_51_P303851 | Olfr646 | NM_147056 | Mus musculus olfactory receptor 646 (Olfr646), mRNA [NM_147056] | -0.74 | -1.67 | 0.010 |
| A_51_P493709 | 2810474C18Rik | AK013405 | Mus musculus 10, 11 days embryo whole body cDNA, RIKEN full-length enriched library, clone:2810474C18 product:hypothetical protein, full insert sequence. [AK013405] | -0.74 | -1.67 | 0.001 |
| A_52_P333097 | Mcoln3 | AK033008 | Mus musculus 12 days embryo male wolffian duct includes surrounding region cDNA, RIKEN full-length enriched library, clone:6720490O21 product:weakly similar to MUCOLIPIDIN [Homo sapiens], full insert sequence [AK033008] | -0.75 | -1.68 | 0.008 |
| A_51_P155458 | A930013K19Rik | BC089590 | Mus musculus RIKEN cDNA A930013K19 gene, mRNA (cDNA clone MGC:107606 IMAGE:6759981), complete cds. [BC089590] | -0.76 | -1.69 | 0.010 |
| A_51_P311379 | Tnrc6a | AK147327 | Mus musculus cDNA, RIKEN full-length enriched library, clone:M5C1013H20 product:trinucleotide repeat containing 6, full insert sequence. [AK147327] | -0.77 | -1.71 | 0.003 |
| A_52_P280668 | 1700001E04Rik | NM_029288 | Mus musculus RIKEN cDNA 1700001E04 gene (1700001E04Rik), mRNA [NM_029288] | -0.78 | -1.72 | 0.002 |
| A_51_P133618 | Clcn1 | X62895 | M.musculus mRNA (cDNA1) skeletal muscle chloride channel, destroyed by insertion of ETn transposon [X62895] | -0.79 | -1.72 | 0.000 |
| A_52_P361007 | NAP054962-1 | NAP054962-1 | Unknown | -0.79 | -1.72 | 0.008 |
| A_52_P12855 | Ankrd43 | NM_183173 | Mus musculus ankyrin repeat domain 43 (Ankrd43), mRNA [NM_183173] | -0.84 | -1.79 | 0.002 |
| A_51_P155873 | Ppp1r3g | XM_127272 | PREDICTED: Mus musculus protein phosphatase 1, regulatory (inhibitor) subunit 3G (Ppp1r3g), mRNA [XM_127272] | -0.89 | -1.86 | 0.003 |
| A_51_P346445 | Hspb7 | NM_013868 | Mus musculus heat shock protein family, member 7 (cardiovascular) (Hspb7), mRNA [NM_013868] | -1.00 | -2.00 | 0.009 |
| A_51_P217498 | Slc2a4 | NM_009204 | Mus musculus solute carrier family 2 (facilitated glucose transporter), member 4 (Slc2a4), mRNA [NM_009204] | -1.25 | -2.39 | 0.001 |
