# Supplementary figures and images for: Post-weaning selenium and folate supplementation affects gene and protein expression and global DNA methylation in mice fed high-fat diets
Source: BMC Med Genomics. 2013 Mar 5;6:7. doi: 10.1186/1755-8794-6-7 (PMC3599545; doi:10.1186/1755-8794-6-7)

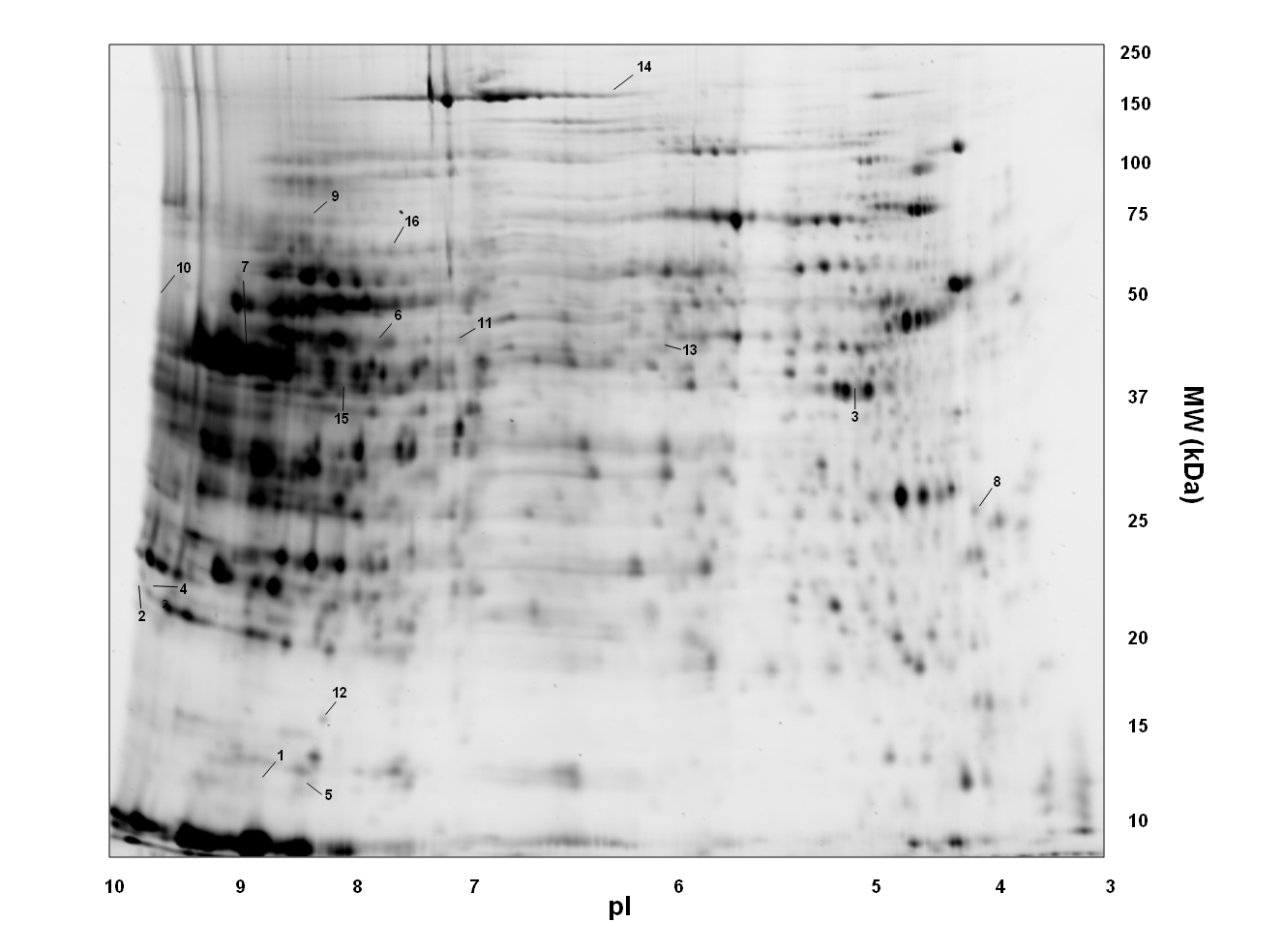

Supplement: Additional file 3: Figure S1 — 2D-DIGE gel representing differentially expressed proteins identified in the liver tissue of female C57 mouse fed a high-fat diet supplemented with adequate selenium and folate (HF-low-suf) born to mothers fed a high-fat diet without supplementation, compared to female offspring maintained on the un-supplemented diet (HF-low-low). Protein annotations are shown in Figure 1 of the main text. The approximate pI and molecular weight (MW) in kDa are given on the x and y axes, respectively. [file 1755-8794-6-7-S3.png]
