# Supplementary material for: Post-weaning selenium and folate supplementation affects gene and protein expression and global DNA methylation in mice fed high-fat diets
Source: BMC Med Genomics. 2013 Mar 5;6:7. doi: 10.1186/1755-8794-6-7 (PMC3599545; doi:10.1186/1755-8794-6-7)
Supplement: Additional file 4: Table S3 — Composition of diets used to determine the effects of post-weaning supplementation with adequate levels of selenium and folate in mice fed high-fat diets. [file 1755-8794-6-7-S4.doc]

## Additional file 4. Composition of diets used to determine the effects of post-weaning supplementation with adequate levels of selenium and folate in mice fed high-fat diets.

|  | **HF-Low, containing 0.4 mg/kg Folate and 0.1 mg/kg of selenium (as analysed)** | **HF-Suf, containing 1.8 mg/kg folate and 0.6 mg/kg selenium (as analysed)** |
| --- | --- | --- |
|  | **% gm** | **% gm** |
| Protein | 20 | 20 |
| Carbohydrate | 49 | 50 |
| Fat | 21 | 21 |
| kcal/gm | 4.68 | 4.68 |
|  |  |  |
| **Ingredient** |  |  |
| Casein | 195 | 195 |
| DL-methionine | 3 | 3 |
| Corn starch | 50 | 50 |
| Maltodextrin 10 | 100 | 100 |
| Sucrose | 341 | 341 |
| Cellulose | 50 | 50 |
| Milk fat, anhydrous | 200 | 200 |
| Corn oil | 10 | 10 |
| Mineral mix S10001^†^ | 0 | 35 |
| Sodium selenite (45.7% Se) | 0.000067 | 0.00146 |
| Mineral mix S19101 (no selenium) | 35 | 0 |
| Calcium carbonate | 4 | 4 |
| Vitamin mix V10001^‡^ | 0 | 10 |
| Vitamin Mix V14901 (no folate) | 10 | 0 |
| Folic acid | 0.00032 | 0.00032 |
| Choline Bitartrate | 2 | 2 |
| Cholesterol, USP | 1.5 | 1.5 |
| Ethoxyquin | 0.04 | 0.04 |
|  |  |  |

^†^S1001: Calcium Phosphate, Dibasic, 29.5% Ca, 22.8% P 500 gm; Magnesium Oxide, 60.3% Mg 24 gm; Potassium Citrate, 1 H2O, 36.2% K 220 mg; Potassium Sulfate, 44.9% K, 18.4% S 52 gm; Sodium Chloride, 39.3% Na, 60.7% Cl 74 gm; Chromium K Sulfate, 12 H2O, 10.4% Cr 0.55 gm; Cupric Carbonate, 57.5% Cu 0.3 gm; Potassium Iodate, 59.3% I 0.01 gm; Ferric Citrate, 21.2% Fe 6 gm; Manganous Carbonate, 47.8% Mn 3.5 gm; Sodium Selenite, 45.7% Se 0.01 gm (not present in S19101); Zinc Carbonate, 52.1% Zn 1.6 gm; Sucrose118.03 gm.

^‡^V10001; Vitamin A Palmitate, 500,000 IU/gm 0.8 gm; Vitamin D3, 100,000 IU/gm 1 gm; Vitamin E Acetate, 500 IU/gm 10 gm; Menadione Sodium Bisulfite, 62.5% menadione 0.8 gm; Biotin, 1.0% 2 gm; Cyancocobalamin, 0.1% 1 gm; Folic Acid 0.2 gm; Nicotinic Acid 3 gm; Calcium Pantothenate 1.6 gm; Pyridoxine-HCl 0.7 gm; Riboflavin 0.6 gm; Thiamin HCl 0.6 gm; Sucrose 978.42 gm.
